# B10 Cells Are Associated With Clinical Prognosis During Adult Symptomatic Acute HBV Infection

**DOI:** 10.3389/fimmu.2022.906650

**Published:** 2022-06-13

**Authors:** Yali Liu, Xiaofei Du, Junfeng Lu, Lina Ma, Yi Jing, Haijing Ben, Xinyue Chen, Jing Zhang

**Affiliations:** ^1^ The Third Unit, Department of Hepatology, Beijing Youan Hospital, Capital Medical University, Beijing, China; ^2^ The First Unit, Department of Hepatology, Beijing Youan Hospital, Capital Medical University, Beijing, China; ^3^ Beijing Institute of Hepatology, Beijing Youan Hospital, Capital Medical University, Beijing, China

**Keywords:** hepatitis B, acute, interleukin-10, regulatory B cell, cytokine, prognosis, B10

## Abstract

There are few reports about the role of B10 cells in acute hepatitis B (AHB) infection. In this study, based on 48 acute hepatitis B infected patients, we analysis the correlation of B10 cells with HBV clinical prognosis. The results showed that B10 was positively correlated with HBsAg and HBeAg and inversely correlated with anti-HBs. The level of B10 in one week before HBsAg clearance was significantly lower than 2 weeks prior to HBsAg clearance and after 1-2 weeks of HBsAg clearance. B10 cell frequency displayed no correlation with HBV DNA; however, it showed significant temporal synchronization with hepatic inflammatory markers such as ALT. B10 level also associated with hospitalization time. These results indicated that B10 is closely related to the clinical prognosis of acute HBV infection.

## Introduction

Although adult acute hepatitis B is mostly self-limited, more than 100,000 people still die from the disease each year, especially in areas with restricted health resources, according to Global Burden of Disease Study (1). There are significant differences in immunological pathogenesis and clinical outcome between acute and chronic HBV infection. For spontaneous clearance of the HBV in the acute setting within several months, both the innate and the adaptive immune system have been proven to be critical (2–4). Otherwise, inflammation will aggravate or turn into chronic persistent infection. The main function of regulatory B cell is to secrete IL-10, so it is also known as B10 cell (5). B10 cells act as an negative regulator and inhibit excessive immune response and maintain homeostasis especially in autoimmune diseases (6). In last two decades, the immunomodulatory effect of B10 on inflammation, autoimmune diseases, and cancer have attracted much attention (7–9). Previous studies were mostly limited to chronic hepatitis B infection (10, 11). However, it is clear that acute hepatitis B virus infection has a more intense dynamics of virological and immunological process. Here, a cohort of patients with acute hepatitis B who made eventually favorable recovery was analyzed and we attempted to elucidate the relationship between B10 cells and the clinical course.

## Materials and Methods

### Study Objects

We conducted a retrospective observational single-center study. The acute hepatitis B (AHB) patients were initially screened according to the Chinese Management Scheme of Diagnostic and Therapy Criteria of Viral Hepatitis criteria (12): Patients needed to meet the following criteria: no history of hepatitis B virus infection (HBsAg negative), presence of clinical symptoms and signs of AHB, significant increase in serum transaminase with or without elevated bilirubin, positive for HBsAg and/or HBeAg and/or HBV DNA, and immunoglobulin M antibody to hepatitis B core antigen positive (IgM anti-HBc). Exclusion criteria were HCV, HDV, HEV, or HIV co-infection. This case series has been described in our previous study (13). Among these cases, we chose the patients with antiviral treatment intervention-free. Overall, there were 48 patients in this study. The blood samples collection was performed between -2 and 24 weeks after the acute hepatitis B diagnosis was confirmed. 24 patients with chronic hepatitis B (CHB) and 10 healthy people (normal controls, NC) as a comparison group. All fresh blood samples were tested immediately or gathering tested within 2 weeks.

This study was guided by the Declaration of Helsinki and the ethical principles developed by the American Human Research Protection Program (AHRPP), and approved by the Ethical Committee of Beijing You An Hospital, Capital Medical University. All patients provided written informed consent.

### HBV Markers

Serologic markers for HBV infection were analyzed by Roche e601 automatic electrochemical luminescence instrument Roche kits (lower limit of detection of HBsAg was 0.05 IU/mL, range from 0.05 to 52,000 IU/mL), according to the manufacturer’s instructions. Quantitation of HBV DNA was performed by polymerase chain reaction using the Roche Cobas/Taqman Real-Time PCR 2.0 System(Roche Diagnostics GmbH, Mannheim, Germany), according to the manufacturer’s instructions (lower limit of detection was 20 IU/mL).

### B10 Confirming

Peripheral blood mononuclear cells (PBMCs) were isolated using lymphocyte separation medium (Ficoll-Hypaque density gradient, Axis-shield, Germany) from 10 ml fresh heparinized blood sample. And then PBMCs were incubated with 1 μM CpG-B ODN2006 (*In vivo*Gen, San Diego, CA, USA) for 96 h at 37°C. PMA (3 ng/ml) and ionomycin (100 ng/ml) were added during the last 4h in the presence of 10 μg/ml brefeldin A (all from Sigma, St Louis, MO, USA). Cells were then surface stained for the markers CD19 Pacific Blue-A, and stained intracellularly with anti-IL-10 APC. So we could assess the ability and the frequency of IL-10 production from purified B cells (14). All antibodies including CD19-PE and IL-10-APC used in flow cytometry were ordered from BD Pharmingen (BD Pharmingen, San Diego, CA, USA). PBMCs were acquired on a FACS Canton flow cytometer (Becton-Dickinson, Franklin Lakes, NJ, USA). FlowJo 7.6.1 (Tree Star Inc., Ashland, OR, USA) was used as the flow analysis software.

### Statistical Analysis

All data were analyzed using SPSS 20.0 (IBM Inc, ArMonk, NY, USA) and the GraphPad Prism 7 software (San Diego, CA, USA). Continuous variables were expressed in mean ± SD or median (interquartile range) as appropriate. Qualitative and quantitative differences between subgroups were analyzed by chi-squared or Fisher’s exact tests for categorical parameters and Student’s t-test or Mann–Whitney test for continuous parameters, paired or unpaired, as appropriate. The Pearson correlation was used to analyze the linear correlation of normally distributed data, otherwise the Spearman rank correction test was used. P < 0.05 was considered statistically significant.

## Results

### Patient Demography and Clinical Outcomes

In total, 48 adult hospitalized patients with AHB were enrolled, which ages ranging from 20 to 71 years (y) The most common chief complaints were dark urine (47/48, 97.9%) scleral or skin jaundice (45/48, 93.8%) and fatigue (42/48, 87.5%). All of the patients displayed significant abnormalities in liver biochemistry enzyme level. The clinical characteristics of all enrolled patients are listed in **Table 1** and the details in **Supplementary Table 1** (**Supplementary 1**). After routine symptomatic treatment and liver protection treatment (but antiviral therapy-free), all 48 patients recovered smoothly. That is, abnormal liver function returned to normal, accompanied by undetectable HBV DNA, and clearance of both HBsAg and HBeAg. At the end of the 48-week follow-up, only 2 patients (4.17%) still did not produce effective Anti-HBs. None of the 48 cases turned chronic.

**Table 1:** Baseline Clinical and Virological Characteristics.

Characteristic	n = 48
**Sex (M/F)**	30/18
**Age (y), mean±SD**	39.6 ± 13.8
**ALT (U/L), median (range)**	1043 (64.1-6367)
**AST (U/L), median (range)**	367 (35.5-3129)
**TBil (μmol/L), median (range)**	83.50 (14.1-379.4)
**HBV DNA (log10 IU/mL) mean±SD**	3.59±1.77
**HBsAg (IU/mL), median (range)**	934.7 (0.05-52000)
**HBeAg (S/CO), median (range)**	6.21 (0.08-1193)
**Transmission Route**	
**sexual**	15 (31.3%)
**tattooing/dental/body piercing**	6 (12.5%)
**healthcare exposure**	2 (4.17%)
**uncertain**	25 (52.1%)
Incubation period^*^ (days), median (range)	37.0 (21-102)
**Average onset day^†^(days), mean±SD**	11.27±6.45

M, male; F, female; y, year; SD, standard deviation; ALT, alanine transaminase; AST, aspartate transaminase; TBIL, total bilirubin.

*Incubation period, depends on data or transmission route available.

^†^Onset day also means the first day of collecting blood sample.

### Analysis of the Correlation of B10 and HBV Markers

**Figure 1:**
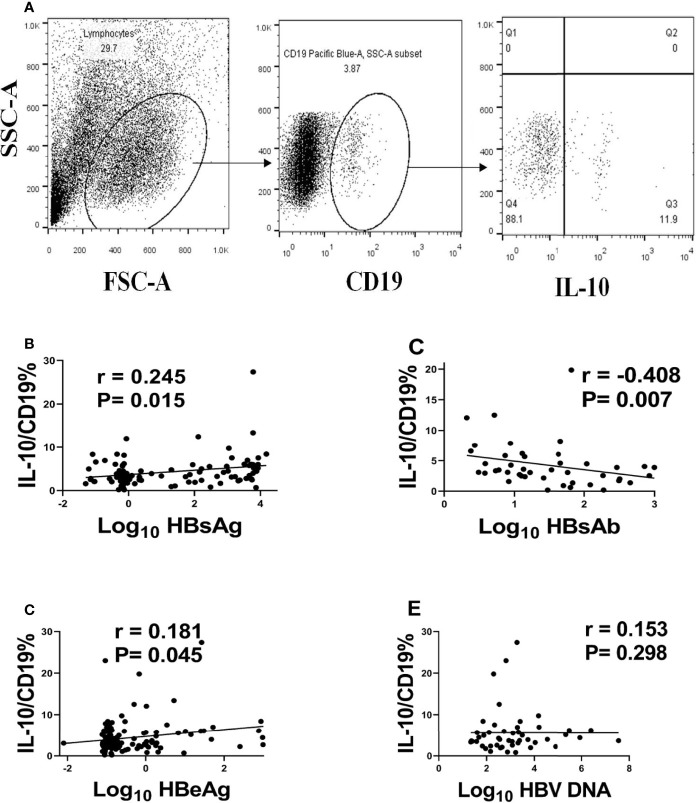
B10 positively associated with HBsAg and HBeAg and negatively associated with anti-HBs. We defined B10 as the level of IL-10 in CD19+ cells **(A)** and analyzed the relationship of B10 with HBsAg **(B)**, anti-HBs **(C)**, HBeAg **(D)** and HBV DNA **(E)**.

In this study, we used CD19 as the marker of B cells and the percentage of IL-10 in Breg or regulatory B cells as the level of B10 (**Figure 1A**). In order to analyze the relationship between B10 and prognosis of acute hepatitis B, we first analyzed the relationship between B10 and HBV DNA, HBsAg, Anti-HBs and HBeAg. We found that B10 was positively correlated with HBsAg (r = 0.245, P = 0.015; **Figure 1B**) and HBeAg (r = 0.181, P = 0.045; **Figure 1D**), and negatively correlated with anti-HBs (r = -0.408, P = 0.007; **Figure 1C**). There was no correlation between B10 and HBV DNA (r = -0.153, P = 0.298; **Figure 1E**).

### Analysis of B10 Level Before and After HBsAg Seroconversion

**Figure 2:**
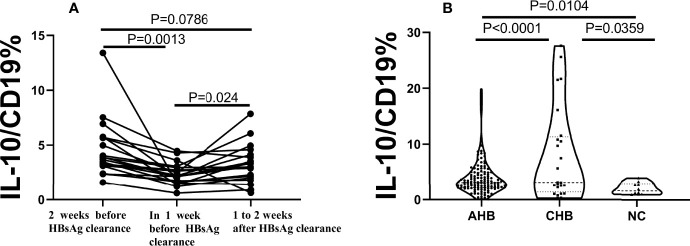
B10 significantly decreased before HBsAg serum clearance in AHB, show significance among the 3 groups. AHB, acute hepatitis **(B)** CHB, chronic hepatitis **(B)** NC, normal control. **(A)**. The dynamics of B10 in each patient with two weeks before HBsAg clearance, one week and within 1-2 weeks after HBsAg clearance. **(B)**. The average of B10 show significance among the 3 groups. the CHB group (n=24) was the highest, followed by the AHB group (n=48), and the lowest was the NC group (n=10).

In order to further analyze the temporal relationship between B10 and the clearance of acute hepatitis B surface antigen. The level of B10 within one and two weeks before HBsAg turned negative and one to two weeks after HBsAg negative were estimated. The level of B10 in one week before HBsAg turning negative was significantly lower than before and after one to two weeks of that (**Figure 2A**). Compared with the chronic hepatitis B (CHB) group and the healthy normal control (NC) group, the CHB group showed the highest B10 level, while the NC group showed the lowest expression level. There were statistical differences among the three groups (**Figure 2B**).

### Correlation Analysis Between Initial B10 Level and Length of Hospital Stay

**Figure 3:**
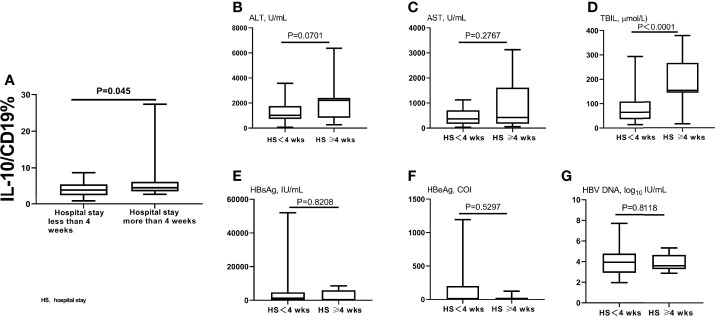
Compared B10 levels and other liver function index between different hospitalization stay time patients. The admitting day’s B10 levels was significant lower in patients within 4 weeks of hospitalization stay than those of more than 4 weeks **(A)**. TBIL levels also show significance between the 2 groups **(D)**, but not ALT **(B)**, AST **(C)**, HBsAg **(E)**, HBeAg **(F)**, and HBV DNA **(G)**.

**Figure 4:**
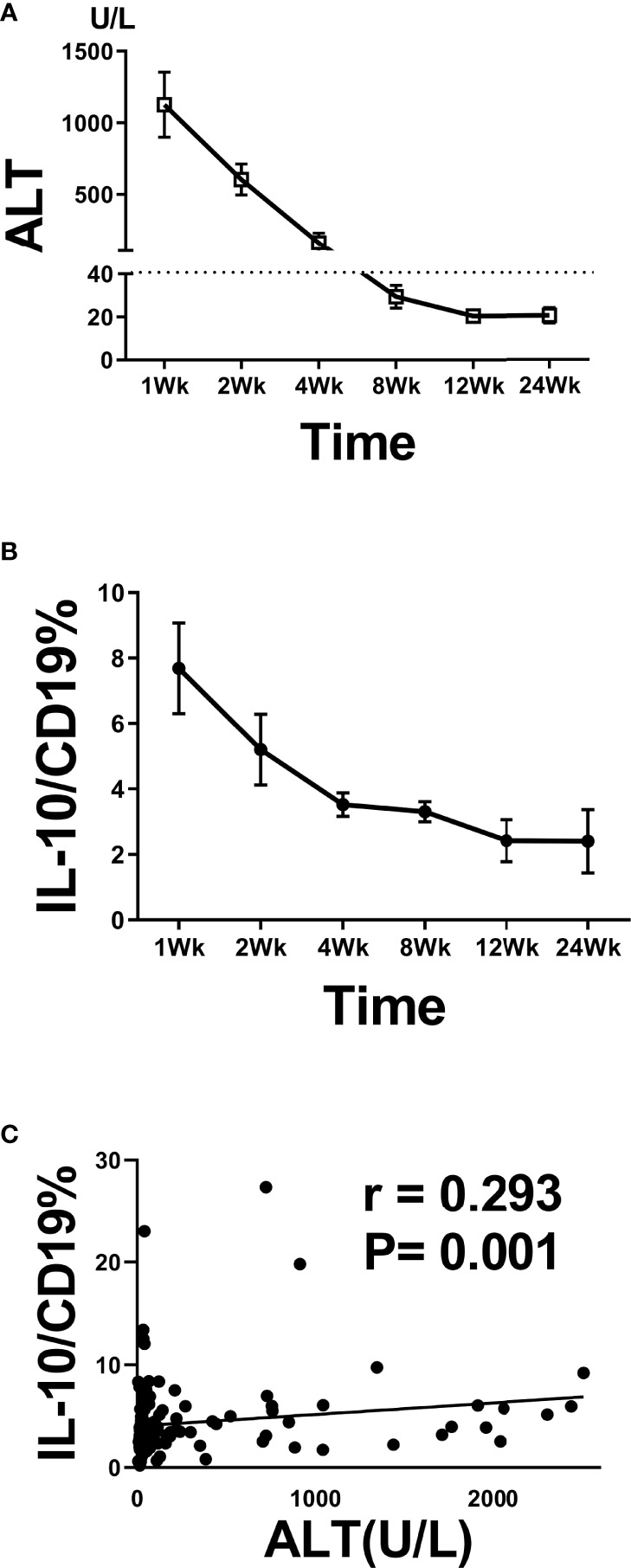
B10 temporal positively associated with ALT during acute HBV infection. Dynamic of ALT **(A)** and B10 **(B)** during hospitalization stay time. The positive correlation between B10 and ALT during hospitalization stay time **(C)**.

We also analyzed the relationship between B10 level at the admission day (i.e., before the HBsAg clearance) and length of hospital stay. The B10 level of patients with hospitalization time more than 4 weeks was higher than that of patients within 4 weeks (**Figure 3A**). Baseline TBIL levels also show significance between the 2 groups (**Figure 3D**), but not ALT, AST, HBsAg, HBeAg, and HBV DNA (**Figures 3B, C, E–G**). The hospitalization time of acute hepatitis B patients are not only related to the time of HBsAg clearance, but also related to the time of liver function recovery. We also analyzed the relationship between B10 and ALT. The levels of ALT (**Figure 4A**) and B10 (**Figure 4B**) gradually decreased with a smooth recovery from the disease. B10 was positively correlated with ALT (r = 0.293, P = 0.001; **Figure 4C**).

## Discussion

Regulatory B cells are a subgroup of B cells. Regulatory B cells are determined by their secretion of negative cytokine IL-10, so the B cells secreting IL-10 are also known as B10. Very little was found in the literature on the question of the role of B10 cells in acute HBV infection. In this study, we found that B10 was closely related to the prognosis of acute HBV by series of samples from 48 cases of acute HBV patients.

The role of B lymphocytes in the pathogenesis of hepatitis B may have been underestimated (15). It is thought that the robust specific T lymphocyte response largely determines the clearance or persistence of HBV infection. Related research also focuses on the depletion mechanisms of HBV-specific T lymphocyte responses (16, 17). B lymphocytes comprise a major part of adaptive immunity, and their main functions in resisting viral infection include a series of events, including complex antigen identification and antigen presentation, as well as the ultimate production of viral antigen-specific antibodies (18). Mizoguchi et al. first coined the term regulatory B cell to describe B cells that suppress disease in a mouse inflammatory bowel disease model (19, 20). B lymphocytes can perform duplex immunomodulation. A prominent example is that B lymphocytes are highly capable of antigen processing and presentation and are necessary for activating CD4+ T lymphocytes. In recent years, attention has been paid to the potential of B lymphocytes in negative immunomodulation, manifested by a number of studies on autoimmune diseases and inflammatory diseases (21–26).

IL-10 is an important inhibitory cytokine in the human body, with a low absolute concentration in peripheral blood. In various microenvironments, it inhibits pro-inflammatory cytokines, Th1 cell responses, and T lymphocyte proliferation, performing multiple negative regulations. In a certain sense, the number of IL-10-producing Breg cells and the IL-10 production level reflect the changes in the body’s inflammation level and immune status. According to a pioneer study by Dr. Maini and her colleagues in 2012, IL-10 levels in plasma and liver inflammation levels of chronic hepatitis B patients were positively correlated, suggesting that the elevation of IL-10 levels is a protective mechanism (10). In terms of the disease course, compared to chronic hepatitis B, acute hepatitis B is characterized by the clinical features of acute onset and self-limiting viral clearance. In acute hepatitis B, inflammation changes rapidly but can eventually recover in a relatively short time. This study revealed the obvious correlation between the B10 frequency and the changes in liver inflammation: the B10 frequency increased significantly when liver inflammation intensified and decreased rapidly to normal during disease recovery. This also fully demonstrates the potential leading role B10 cell plays in regard to inflammation and immunity.

As stated above, because the secretion of IL-10 is not limited to a particular cell or cell subset, B10 cells may also comprise different cell subsets and may have certain heterogeneities. Indeed, it significantly increased at the peak of inflammatory activities and decreased when inflammation was eased, suggesting that it can inhibit inflammation. The inflammation levels of acute hepatitis B can be self-limiting, synchronically related to the levels of B10 frequency, indicating the latter’s function in inhibiting overdevelopment of inflammation to prevent spread. In the AHB recovery phase, B10 drop to a low level and prevent the disease from turning into a chronic persistent infection. Even if the P values have statistical significance, the absolute values of Pearson correlation coefficient (r) between B10 cell and HBsAg, HBeAg and ALT are not strong enough. A larger sample size to verify the correlation is expected. It is no doubt that regulatory T cells can perform negative regulation by means of IL-10, and this has been confirmed for chronic hepatitis B. B10 cells exerted dysregulation in T cell function through IL-35 dependent mechanism and depend on cell-to-cell contact style (27, 28).

With AHB standard management procedure and no antiviral intervention, we could conclude that the length of hospital stay is directly related to severity of illness. In this study, the frequency of B10 cell was higher in patients hospitalized for longer than 4 weeks than in patients hospitalized for less than 4 weeks. These results further suggest that B10 is positively correlated with the course and prognosis of acute liver inflammatory disease. Before and after HBsAg clearance, we also clearly observed the critical time point which the expression of B10 frequency is the lowest. Accompanied by HBsAg clearance and anti-HBs appearance gradually, B10 return to the previous normal level. These regulatory cells are also regulated by regulators and may be related to IL-35 or IL-21 (8), which needs to be confirmed by further studies.

Previous study revealed that the depletion of Treg cells in the PBMCs of patients with chronic hepatitis B did not alter the frequency of B10 cells or their ability to produce IL-10 (11), indicating regulatory T or B lymphocyte have their own unique pathway to influence immune response. Also, there are other IL-10-producing immune cells such as T cell, monocytes and NK cells, not involved in this assay with regret (29–31). We speculate that these immune cells focus on secreting IL-10, forming a complex and interacting regulatory network, and exert inhibitory effects organically. Furthermore, the lack of acute fulminant hepatitis and liver specimens is the deficiency of this study. Additionally, the development about HBsAg-specific and/or HBcAg-specific B cells maybe novel targets for antiviral strategies toward functional cure of chronic HBV infection attracted more attentions (32–34). The failure to explore the phenotype features of those HBV-specific B cells in the environment of acute hepatitis B is also one of the short-comings of this study.

In conclusion, we found that B10 is closely related to the clinical prognosis of acute HBV infection. Whether B10 is one of the pathogenic mechanisms of acute fulminant hepatitis and whether intervention of B10 cells is helpful to the elimination of chronic HBV deserves further study.

## Data Availability Statement

The original contributions presented in the study are included in the article/**Supplementary Material**. Further inquiries can be directed to the corresponding authors.

## Ethics Statement

The studies involving human participants were reviewed and approved by Ethical Committee of Beijing YouAn Hospital, Capital Medical University. The patients/participants provided their written informed consent to participate in this study.

## Author Contributions

YL, XC, and JZ conceived and designed the experiments; YL and XD performed the experiments; YL and HB analyzed the data; XD, LM, JL, and YJ contributed patients recruiting and management. YL and JZ wrote the paper. All authors contributed to the article and approved the submitted version.

## Funding

Thirteenth Five-Year Major Science and Technology Projects (2017ZX10202202-005-010), the Capital Health Research and Development Projects (2020-1-3011). 2022 Research project on education and teaching reform of Capital Medical University (2022JYY260).

## Conflict of Interest

The authors declare that the research was conducted in the absence of any commercial or financial relationships that could be construed as a potential conflict of interest.

## Publisher’s Note

All claims expressed in this article are solely those of the authors and do not necessarily represent those of their affiliated organizations, or those of the publisher, the editors and the reviewers. Any product that may be evaluated in this article, or claim that may be made by its manufacturer, is not guaranteed or endorsed by the publisher.
