# Supplementary material for: B10 Cells Are Associated With Clinical Prognosis During Adult Symptomatic Acute HBV Infection
Source: Front Immunol. 2022 Jun 13;13:906650. doi: 10.3389/fimmu.2022.906650 (PMC9234142; doi:10.3389/fimmu.2022.906650)
Supplement: Supplementary file 1 [file Table_1.pdf]

Supplementary Table 1

The baseline demography and clinical characters of all 48 patients

Patient no.	sex	Age (y)	ALT (U/L)	AST (U/L)	TBil (μmol/L)	HBsAg (IU/mL)	Anti-HBs (IU/mL)	HBeAg (COI)	Anti-HBc IgM (COI)	HBVDNA (IU/mL)	LOHS (days)
**01**	M	38	1784.6	758.5	27.4	4647	<2	799.4	21.85	4.76	17
**02**	M	30	2048.7	1058.5	166.7	1471	<2	14.09	9.99	3.01	13
**03**	M	45	220.3	120.7	16.4	19.91	<2	1.83	11.26	NA	10
**04**	M	53	909.6	179.3	109	0.507	<2	0.081	2.19	NA	19
**05**	M	27	271.2	58.7	16.5	5910	<2	0.281	12.32	NA	28
**06**	M	29	920.5	151.4	151.9	35.63	<2	0.26	22.09	3.61	33
**07**	F	20	1043.9	512.9	39.4	3252	<2	759.4	15.02	6.4	20
**08**	M	46	6367.1	3128.8	178.3	0.63	<2	1.17	6.79	NA	38
**09**	M	24	1764.4	645.1	92.7	5985	50.89	47.21	12.80	4.55	18
**10**	F	42	756.6	169.1	28.5	8445	<2	25.24	13.22	2.7	27
**11**	M	38	2441.7	1125.8	89	87.99	<2	10.19	8.60	2.74	16
**12**	F	29	723.3	197.3	145.7	6007	287.7	23.13	10.19	3.28	39
**13**	F	26	1533	749.5	127.2	555.75	<2	858.2	22.8	NA	24
**14**	M	34	2210.3	976.9	159	8586	8.9	26.18	13.72	3.45	30
**15**	M	66	1014.2	755.1	76.7	4257	<2	48.032	8.09	5.47	25
**16**	F	39	522	86.5	19.7	152.3	<2	37.1	15.28	4.78	10
**17**	M	37	2060.4	788	43	4617	<2	200.2	3.29	3.34	16
**18**	M	51	1345.1	368.2	65.9	1104	<2	1193	18.81	4.18	18
**19**	M	48	2511	1383.4	153.7	19.78	<2	0.14	26.71	2.88	36
**20**	M	34	385	35.5	45.3	307.6	<2	1.86	18.42	3.22	9
**21**	M	27	728.9	111.4	39.6	4038	<2	0.241	24.59	4.2	15
**22**	F	20	3574.5	562.6	150.8	10.46	<2	2.06	16.72	4.06	27
**23**	F	54	909.3	366.8	46	7556	<2	1.07	17.04	3.83	18
**24**	F	54	444.1	244.3	18.2	142.6	<2	51.41	26.15	2.51	10
**25**	F	55	812.6	605.8	26.3	183.2	<2	12.79	16.20	4.74	9
**26**	M	29	1246.3	198.6	129.5	7681	50.89	0.657	14.79	4.61	11
**27**	M	24	1631.3	582.9	97.5	82.24	<2	0.133	12.76	4.17	11
**28**	F	48	754.1	808.6	293.7	9385	<2	47.98	6.44	5.64	23
**29**	M	50	1957.3	464.3	39.5	0.05	292.5	5.26	7.69	3.73	9
**30**	F	22	2039	710.4	108.7	0.07	185.8	0.408	11.49	3.08	13
**31**	M	52	523.2	70	55.8	765.3	<2	0.133	13.24	3.5	9
**32**	M	44	852.2	169.7	52.7	7592	<2	0.356	10.30	5.94	12
**33**	M	23	1042.1	151.1	86.8	0.918	312.7	0.145	14.17	1.96	10
**34**	M	36	759	102.6	64.4	285.2	<2	1.03	8.89	3.11	17
**35**	M	28	3024	1044.4	129.9	0.09	11.72	0.14	13.58	2.79	26
**36**	F	22	1437.7	214.1	81.9	2133	<2	0.15	18.68	4.9	15
**37**	F	34	995.2	238.2	109.4	4126	25.93	956.7	15.17	2.88	22
**38**	M	66	1058.4	320.2	142.3	203.9	<2	0.134	26.84	4.67	30
**39**	F	58	64.1	144.1	14.1	2414	<2	890.5	2.09	7.13	10
**40**	M	70	1623.9	317.4	116.1	0.05	88.35	0.89	14.42	2	27
**41**	F	36	1710.7	588.3	85.1	1703	<2	252.9	20.61	4.04	14
**42**	F	32	597.4	177.7	23.4	44.77	<2	2.14	5.09	2.17	13
**43**	M	48	722.5	276.3	45.9	0.05	17.39	3.01	14.57	3.32	9

**44**	F	31	2308	1855	356	1326	<2	122.5	10.83	5.34	32
**45**	M	71	1000.3	702	28.6	8397	<2	332.1	8.19	7.71	17
**46**	F	25	1912.1	909.8	228	52000	<2	1027.6	18.27	5.49	26
**47**	M	38	705.6	118.5	36.4	5212	<2	118.4	21.96	2.44	13
**48**	M	46	2282.9	423.3	379.4	1.06	35.12	7.16	24.80	4.05	41

（LOHS, length of hospital stay）
